# Primitive Sjögren’s Syndrome Revealed by Bilateral Pleurisy in a Teenager: An Unexpected Case

**DOI:** 10.7759/cureus.84514

**Published:** 2025-05-20

**Authors:** Meriem Rhazari, Ikram Sadki, Hiba Ramdani, Afaf Thouil, Hatim Kouismi

**Affiliations:** 1 Pulmonology, Faculty of Medicine and Pharmacy of Oujda, Mohammed VI University Hospital, Mohammed First University, Oujda, MAR; 2 Internal Medicine, Faculty of Medicine and Pharmacy of Oujda, Mohammed VI University Hospital, Mohammed First University, Oujda, MAR; 3 Medicine, Faculty of Medicine and Pharmacy of Oujda, Mohammed VI University Hospital, Mohammed First University, Oujda, MAR

**Keywords:** anti-ssa/ro antibodies, auto immune disease, autoimmunity, bilateral pleurisy, primitive sjögren's syndrome

## Abstract

Primary Sjögren's syndrome (PSS) is a rare autoimmune disease in children and adolescents. The initial pleural manifestations are exceptional. We report the case of a 16-year-old adolescent hospitalized for bilateral chest pain with pleurisy. The initial diagnosis of tuberculosis was suspected due to the clinical context and local endemicity, but microbiological and histological examinations were negative. The presence of anti-Sjögren's syndrome type A (anti-SSA) antibodies and a salivary gland biopsy confirming chronic sialadenitis allowed for the diagnosis of PSS. Management by corticosteroid therapy resulted in a significant clinical improvement.

Although rare, PSS should be considered in the differential diagnosis of lymphocytic pleurisy, especially when anti-infective treatments fail. Pleuropulmonary manifestations are poorly described and can delay the diagnosis. This report highlights the diversity of presentations of PSS and the importance of a multidisciplinary approach for early diagnosis, allowing for improved prognosis of systemic complications.

## Introduction

Gougerot-Sjögren syndrome (GSS) is an autoimmune disease that mainly affects the exocrine glands, especially salivary and lacrimal glands [1]. It is considered primary (primary Sjögren's syndrome (PSS)) when not associated with other autoimmune diseases and secondary (secondary Sjögren's syndrome) when linked to clearly defined systemic pathologies, such as rheumatoid arthritis, systemic lupus, scleroderma, or mixed connective tissue disease. It can also be associated with other organ-specific autoimmune diseases, such as autoimmune thyroiditis and primary biliary cirrhosis [2].

Sjögren's syndrome frequently affects middle-aged women, with an estimated prevalence of about 0.5% of the general population. Although it is less common in children and adolescents, cases have been reported [3]. It is important to note that the diagnosis of Sjögren's syndrome in young individuals might be difficult due to symptom diversity and the disease's rarity in this age group [3]. Pulmonary manifestations of Sjögren's syndrome, which affect approximately 9% to 20% of patients, include airway abnormalities, interstitial lung diseases (ILD), and lymphoproliferative disorders [4], while pleural involvement remains rare and poorly documented in the literature [5]. We report a case of PSS in a 16-year-old adolescent, revealed by pleurisy, which is exceptional and underlines the importance of considering this pathology in the differential diagnosis of pleurisy.

## Case presentation

**Figure 1:**
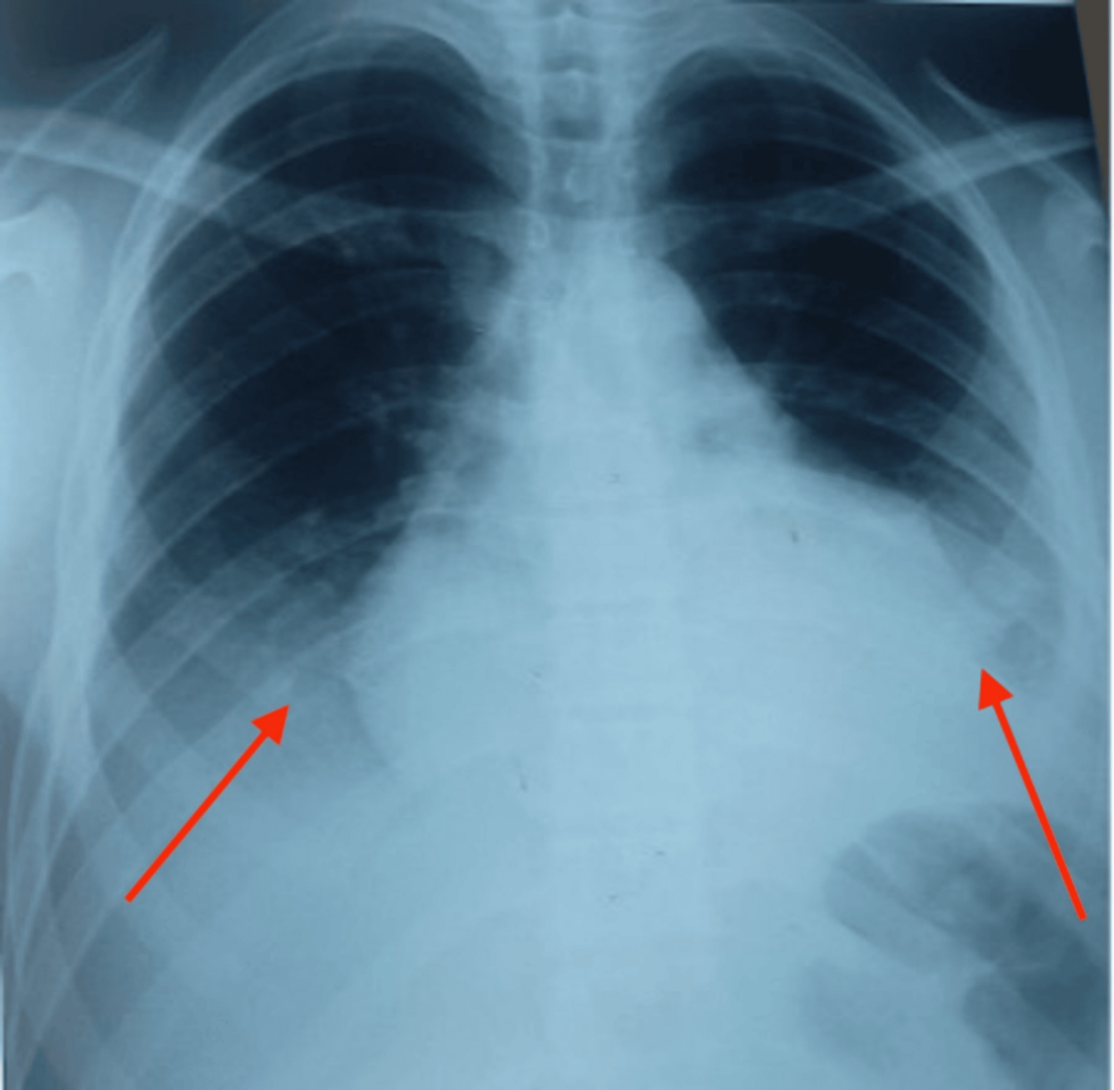
The chest X-ray showing bilateral heterogeneous opacity occupying the lower third of both thoracic hemifields (red arrows), with cardiomegaly.

We report the case of a 16-year-old adolescent with no significant medical history, admitted for the management of acute bilateral basithoracic pleuritic chest pain accompanied by unquantified fever, night sweats, asthenia, and anorexia. On clinical examination, the patient exhibited oxygen desaturation to 87% on room air, which improved to 93% with 3 L of supplemental oxygen. The patient was febrile at 38.3°C, tachycardic at 122 bpm, and presented with profuse sweating and bilateral pleural effusion syndrome. The remainder of the physical examination was unremarkable. A chest X-Ray (Figure 1) demonstrated heterogeneous opacities occupying the lower two-thirds of both hemithoraces, along with cardiomegaly. Cardiac ultrasound revealed a moderate pericardial effusion with fibrin deposition, with normal left ventricular (LV) function.

**Figure 2:**
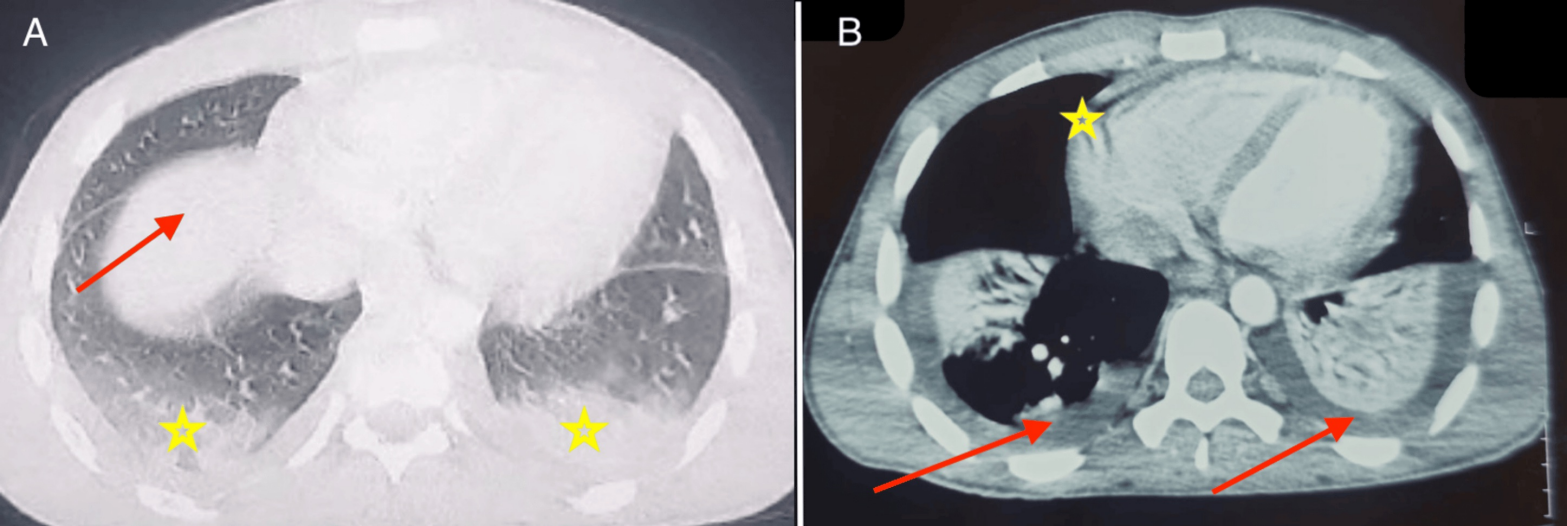
Axial view of contrast-enhanced thoracic CT scan A: Parenchymal window shows ground-glass hyperdensity in the right upper lobe (red arrow) with bilateral lower lobe pulmonary consolidation (yellow stars); B: Mediastinal window shows moderate bilateral pleural effusion (red arrows) with pericardial effusion (yellow star)

An ultrasound-guided pleural puncture yielded citrine yellow fluid with a lymphocyte predominance of 75%. Bacteriological studies and testing for acid-fast bacilli (direct examination and Xpert MTB/Rif) were negative. Pleural biopsy showed non-specific inflammatory changes. The diagnosis of pleural and pericardial tuberculosis was made based on clinical criteria. Adenosine deaminase levels in the pleural fluid were slightly elevated at 34, and blood quantiferon testing was negative. The patient was initiated on anti-tubercular therapy with corticosteroids. Over one week, the patient showed clinical improvement, with oxygen saturation increasing to 95% on room air and resolution of chest pain. On the 7th day, the patient experienced a recurrence of chest pain accompanied by tachycardia at 137 bpm, prompting a thoracic CT angiogram (Figure 2). A non-contrast thoracic CT scan was performed to rule out lymphoma, yielding unremarkable findings.

**Figure 3:**
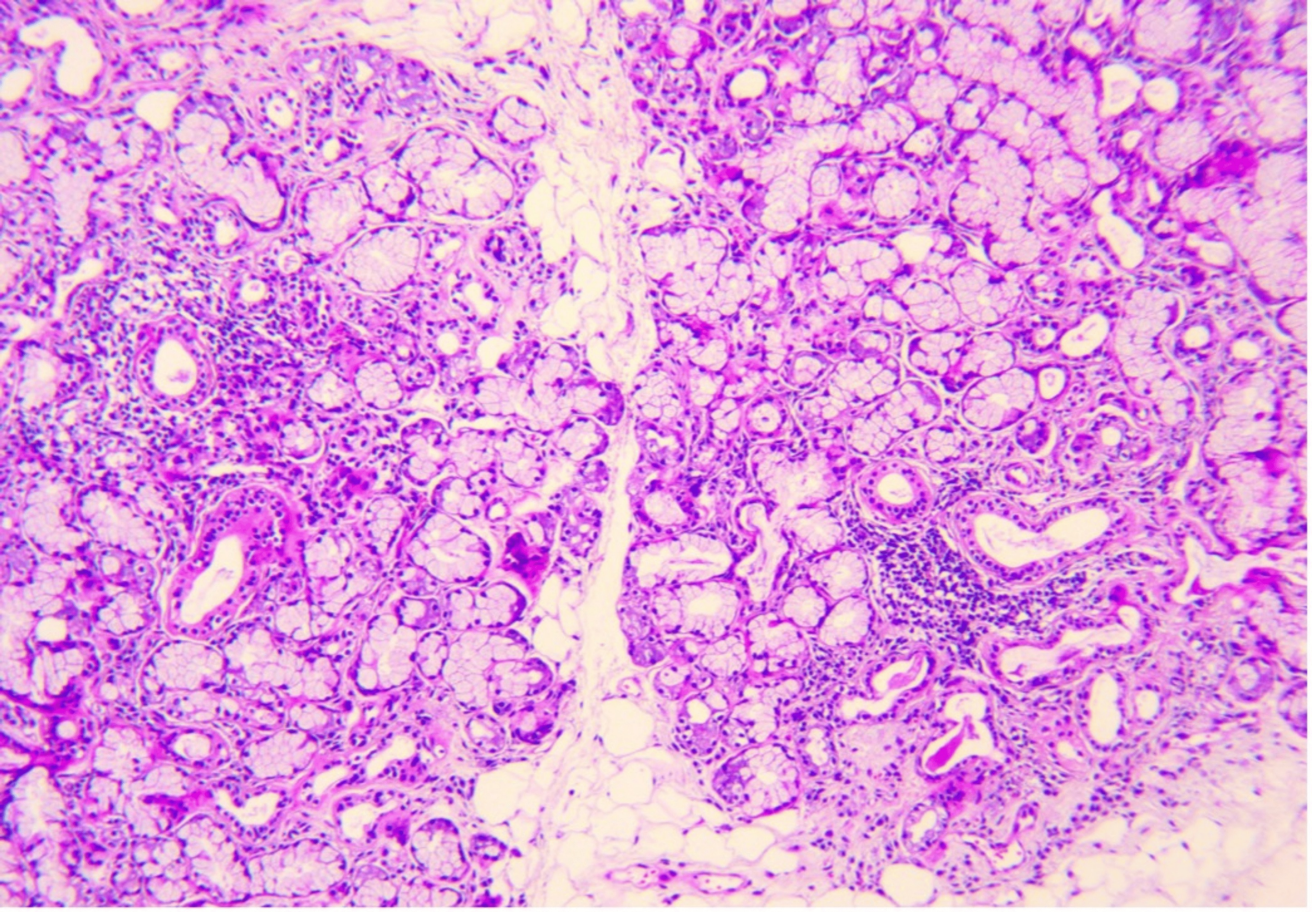
A high-magnification histological image revealing stage IV sialadenitis.

Further investigations revealed positive antinuclear antibodies with anti-Sjögren's syndrome type A (anti-SSA) antibodies, and a biopsy of the accessory salivary glands demonstrated chronic sialadenitis classified as stage IV (Figure 3), leading to the diagnosis of PSS. The patient was initially treated with corticosteroids (prednisone) at a dose of 1 mg/kg/day, planned for eight weeks with a tapering schedule starting at day 15. However, the treatment was continued in combination with azathioprine as background therapy for GSS. The patient is currently maintained on hydroxychloroquine (Plaquenil), with clinical improvement.

In the case of lymphocytic pleurisy in an adolescent living in a country endemic to tuberculosis, the differential diagnosis mainly includes pleural tuberculosis. However, the diagnostic tests, including Xpert mycobacterium tuberculosis complex (MTBC)/resistance to rifampin (RIF), adenosine deaminase (ADA) activity, and the Quantiferon test, were all negative, and no clinical improvement was observed under anti-tuberculosis treatment. Lymphoma was excluded based on normal blood tests, a thoracic-abdominal and pelvic CT scan without lymphadenopathy or mass, as well as the absence of neoplastic cells in the pleural fluid and a negative pleural biopsy.

## Discussion

Primary Sjögren's syndrome was first described in 1926 by Gougerot [6]. It predominantly affects middle-aged women and may present with various systemic manifestations. Although pulmonary involvement is relatively uncommon, it can occasionally include pleural effusion, a rarely reported feature in the literature [7]. Recently, an increased risk of pulmonary involvement has been identified in the male sex (even though Sjögren's syndrome remains more frequent in women), smokers, older age, having higher hypergammaglobulinemia, and the presence of rheumatoid factor, although these criteria are not very sensitive [8].

Juvenile-onset Sjögren’s syndrome is rare and often underdiagnosed due to its variable clinical presentation and the lack of validated pediatric diagnostic criteria, making its true prevalence in the pediatric population difficult to determine [3]. Gougerot-Sjögren syndrome is extremely rare in childhood; however, pulmonary involvement in the form of lymphoid interstitial pneumonia (LIP) has been reported in an exceptional case at the age of 14 [9]. In the literature, several studies have demonstrated that the age of onset influences the clinical-biological expression, the evolutionary pattern, and the prognosis of autoimmune diseases, such as systemic lupus erythematosus and rheumatoid arthritis [10].

Gougerot-Sjögren syndrome occurs in individuals genetically predisposed to autoimmunity with a high prevalence of HLA B8 and DR3 haplotypes [11,12]. The pathophysiology of Sjögren's syndrome remains complex and poorly understood, resulting from an abnormal activation of the immune system where the epithelial cells of the salivary glands are attacked by T and B lymphocytes, causing inflammation and tissue destruction. The B lymphocytes, particularly through the B-cell activating factor (BAFF), play a central role in this pathogenesis. Pleurisy can occur due to systemic inflammation, with autoantibodies such as anti-SSA/Ro inducing serous membrane damage. Although rare, pleural involvement remains possible through local activation of inflammatory cells [11,12]. To date, few cases of PSS complicated by pleural effusion have been described, among which pleural effusion exceptionally constituted an inaugural manifestation [13].

A few cases of PSS associated with pleural effusion have been reported, but without detailed descriptions of these cases or the diagnostic methods used. Pleural effusions in GSS are often bilateral and may be associated with symptoms such as dyspnea and chest pain. Effusion may develop slowly, sometimes leading to a delay in diagnosis. It is frequently lymphocytic, with a predominance of T cells and the presence of anti-SSA and anti-Sjögren's syndrome type B (anti-SSB) antibodies detected in pleural fluid as well as in serum. This procedure allows for the distinction of autoimmune pleurisy from other causes, such as infection or tumors [14]. Diagnostic criteria [15,16] are proposed by the 2017 European League Against Rheumatism/American College of Rheumatology (EULAR/ACR) classification, adapted from Shiboski et al. (Table 1).

**Table 1:** The 2017 EULAR/ACR classification for Sjögren's syndrome This classification has been adapted from Shiboski et al. [15]. EULAR/ACR: European League Against Rheumatism/American College of Rheumatology; anti-SSA: Anti-Sjögren's syndrome type A

Criteria	Score
Lymphocytic sialadenitis with a focus score ≥ 1 on accessory salivary gland biopsy and a focus score ≥ 1 foci/4mm²	3 points
Positive anti-SSA/Ro antibodies*	3 points
Ocular staining score ≥ 5 (or van Bijsterveld score ≥ 4) in at least one eye	1 point
Schirmer test ≤ 5 mm/5 min in at least one eye	1 point
Unstimulated salivary flow ≤ 0.1 mL/min	1 point

Given the rarity of this event, the therapeutic management of these pleural effusions is not well defined due to the limited number of specific studies on the subject. According to an article published in the journal La Revue de Médecine Interne, the pleuropulmonary manifestations of GSS may include pleural effusions, and the treatment mainly relies on the use of corticosteroids to reduce inflammation. In refractory cases, immunosuppressants may be considered [17].

## Conclusions

This rare case of PSS revealed by bilateral pleurisy in an adolescent highlights the importance of considering this pathology in the diagnosis of lymphocytic pleurisies, even in the context of tuberculous endemicity. An early diagnosis, guided by autoimmune serology and salivary gland biopsy, allows for appropriate management. Corticosteroid therapy remains the cornerstone of treatment, although pleural manifestations require further studies for optimal recommendations.
